# The risk of herpes zoster is positively associated with obesity, especially morbid obesity

**DOI:** 10.1038/s41598-024-65195-x

**Published:** 2024-06-21

**Authors:** Hsiao-Lan Chen, Chia-Hung Chen, Wen-Che Hsieh, Yu-Han Huang, Tzu-Ju Hsu, Fuu-Jen Tsai, Yung-Chi Cheng, Chao-Yu Hsu

**Affiliations:** 1 Division of Respiratory Therapy, Ditmanson Medical Foundation, Chia-Yi Christian Hospital, Chia-Yi, Taiwan; 2 Department of Medical Education, Ditmanson Medical Foundation, Chia-Yi Christian Hospital, Chia-Yi, Taiwan; 3 Department of Medical Imaging, Ditmanson Medical Foundation, Chia-Yi Christian Hospital, Chia-Yi, Taiwan; 4 Department of Chinese Medicine, Ditmanson Medical Foundation, Chia-Yi Christian Hospital, Chia-Yi, Taiwan; 5 Management Office for Health Data, Clinical Trial Research Center, China Medical University Hospital, Taichung, Taiwan; 6 School of Chinese Medicine, College of Chinese Medicine, China Medical University, Taichung, Taiwan; 7 Department of Medical Research, China Medical University Hospital, Taichung, Taiwan; 8 Division of Medical Genetics, China Medical University Children’s Hospital, Taichung, Taiwan; 9 Department of Biotechnology and Bioinformatics, Asia University, Taichung, Taiwan; 10 Department of Rehabilitation, Ditmanson Medical Foundation Chia-Yi Christian Hospital, Chia-Yi, Taiwan; 11 Department of Artificial Intelligence and Healthcare Management, Central Taiwan University of Science and Technology, Taichung, Taiwan; 12 Center for General Education, National Taichung University of Science and Technology, Taichung, Taiwan; 13 Department of General Education, National Chin-Yi University of Technology, Taichung, Taiwan

**Keywords:** Obesity, Herpes zoster, Morbid obesity, Weight reduction, Infectious diseases, Metabolic disorders, Skin diseases

## Abstract

This study aimed to investigate the association between obesity and herpes zoster (HZ) occurrence. This study used data covering 2 million people in Taiwan in 2000, which were obtained from the National Health Insurance Research Database. The cohort study observed aged 20–100 years with obesity from 2000 to 2017 (tracking to 2018). Obesity was indicated by the presence of two or more outpatient diagnoses or at least one admission record. And, obesity was categorized into non-morbid obesity and morbid obesity. Patients with HZ before the index date were excluded. The obesity cohort and control cohort were matched 1:1 according to age, sex, comorbidities, and index year. There were 18,855 patients in both the obesity and control cohorts. The obesity cohort [adjusted hazard ratio (aHR) 1.09] had a higher risk of HZ than the control cohort. Further analysis, the morbid obesity group (aHR 1.47), had a significantly higher risk of HZ than the non-morbid obesity group. Among the patients without any comorbidities, the patients with obesity had a significantly higher risk of developing HZ than the patients without obesity (aHR 1.18). Obese patients are at a higher risk of HZ development, especially in the patients with morbid obesity. Weight reduction is critical for preventing the onset of chronic diseases and decreasing the risk of HZ in patients with obesity.

## Introduction

According to the World Health Organization, obesity is defined as having a body mass index (BMI) of more than 30 kg/m^2^. In Taiwan, the Ministry of Health and Welfare has established the following guidelines for obesity based on body mass index (BMI). A BMI of 27 kg/m^2^ or higher is considered the threshold for obesity, while a BMI exceeding 40 kg/m^2^ is classified as morbid obesity. These criteria help assess individuals' weight status and provide a reference for healthcare professionals in determining appropriate interventions or treatments.

In 1975, the global prevalence of obesity was less than 1%; however, it increased to 6%–8% in 2016^1^. Between 2017 and 2018, the prevalence of obesity and severe obesity in adults was 42.4% and 9.2%, respectively, in the United States. In addition, adults aged 40–59 years had the highest prevalence of severe obesity^2^. In southern China, the prevalence of overweight and obesity was 25.8% and 7.9%, respectively^3^. A meta-analysis of 288 studies covering 13.2 million individuals found that the global prevalence of central obesity was 41.5%. The prevalence of central obesity was higher among older adults, women, urban dwellers, Caucasians, and individuals from high-income countries. Moreover, the highest prevalence of central obesity was 55.1% and 52.9% in Sothern America and Central American, respectively^4^. Because obesity is a risk factor for chronic diseases, Keramat et al. suggested that strategies for weight management should be incorporated into health promotion programs to prevent chronic diseases^5^.

Herpes zoster (HZ) is caused by the reactivation of varicella-zoster virus (VZV) and presents as painful vesicles with a dermatomal distribution. A systematic review of 69 studies found that the incidence of HZ was between 5.23 and 10.9 per 1000 person-years worldwide. In addition, the authors reported that women (6.05–12.8 per 1000 person-years) had a higher incidence than men (4.3–8.5 per 1000 person-years)^6^. Immunocompromised patients have a higher risk of HZ. Schröder et al. found that the incidence of HZ in immunocompromised and immunocompetent patients was 11.5 and 5.9 per 1000 person-years, respectively, indicating that HZ incidence is nearly double in immunocompromised patients compared with immunocompetent patients^7^.

Mehta et al. reported that only 1 of 112 saliva samples from eight astronauts was positive for VZV DNA before space flight. However, 61 of 200 samples were positive for VZV DNA during and after spaceflight. These findings indicate that VZV can be reactivated by stress^8^. In our previous study, we found that stress from disease was associated with HZ development. Musculoskeletal chronic pain^9–14^, which causes stress to affected individuals, has been associated with HZ development. In addition, endocrine disease, namely polycystic ovary syndrome (PCOS)^15^, also increases risk of HZ occurrence. To date, whether obesity is a risk factor for HZ reactivation remains unknown. This study investigated the association between obesity and HZ occurrence.

## Methods

### Data source

The National Health Insurance Research Database (NHIRD) contains health insurance claims data for 99% of Taiwanese residents. This study used a sample data from the NHIRD covering a population of 2 million in 2000. Disease diagnoses were coded according to the *International Classification of Diseases, 9th Revision and 10th Revision, Clinical Modification* (*ICD-9-CM and ICD-10-CM*).

### Study population, outcome and comorbidities

This cohort study observed patients aged 20–100 years with obesity from 2000 to 2017 (tracked to 2018). Obesity (*ICD-9-CM: 278.0; ICD-10-CM: E66*) was indicated by the presence of two or more outpatient diagnoses or at least one admission record. To assess the impact of obesity severity, we categorized obesity into two groups: non-morbid obesity (*ICD-9-CM: 278.00; ICD-10-CM: E66.09, E66.1, E66.8, E66.9*) and morbid obesity (*ICD-9-CM: 278.01; ICD-10-CM: E66.01, E66.2*). The index date was defined as the date when obesity was diagnosed, and patients with HZ (*ICD-9-CM: 053; ICD-10-CM: B02*) before the index date were excluded. The obesity and control cohorts underwent 1:1 propensity score matching based on age, sex, comorbidities, and index year. The covariates were comorbidities, such as diabetes mellitus (DM) (*ICD-9-CM: 250; ICD-10-CM: E08–E13*), chronic kidney disease (CKD) (*ICD-9-CM: 585; ICD-10-CM: N18.4–N18.9*), coronary artery disease (CAD) (*ICD-9-CM: 410–414; ICD-10-CM: I20–I25*) and cancer (*ICD-9-CM: 140–208; ICD-10-CM: C*).

### Statistical analysis

In this analysis, the frequencies and percentages of age, sex, and comorbidities as variables are presented. To compare the differences between categorical and continuous variables in the two groups, a chi-square test and t-test were employed, respectively. The variables of age and follow-up period are presented as mean and ± standard deviation (SD). To determine the incidence rate (IR) and hazard ratios (HR) of HZ, we utilized multivariable Cox proportional hazards models that were adjusted for age, sex, and comorbidities. Furthermore, we applied the Schoenfeld Individual Test to assess the proportional hazards assumption, similar to how the Shapiro–Wilk test evaluates normality. If the p-value is less than 0.05, it indicates a violation of the assumption. In our analysis, the Schoenfeld test yielded a p-value of 0.07, which is greater than 0.05, confirming that there is no violation of the proportional hazards assumption. We used the Kaplan–Meier method to derive the cumulative incidence curve of obesity and fitted the curve using the log-rank test. A two-sided *p*-value less than 0.05 was considered significant. All statistical analyses were performed using SAS, version 9.4 (SAS Institute Inc., Cary, NC). A graph of the cumulative incidence of obesity was obtained using R Studio.

### Ethics approval and consent to participate

The study was conducted according to the Declaration of Helsinki and it was approved by the institutional review board of Ditmanson Medical Foundation, Chia-Yi Christian Hospital, Chia-Yi, Taiwan (approval number: IRB2022115, date of approval: December 19, 2022). Because the NHIRD was used for this study and the patients’ information has been de-identified, the informed consent was waived by the Institutional Review Board of Ditmanson Medical Foundation, Chia-Yi Christian Hospital, Chia-Yi, Taiwan.

## Results

**Figure 1:**
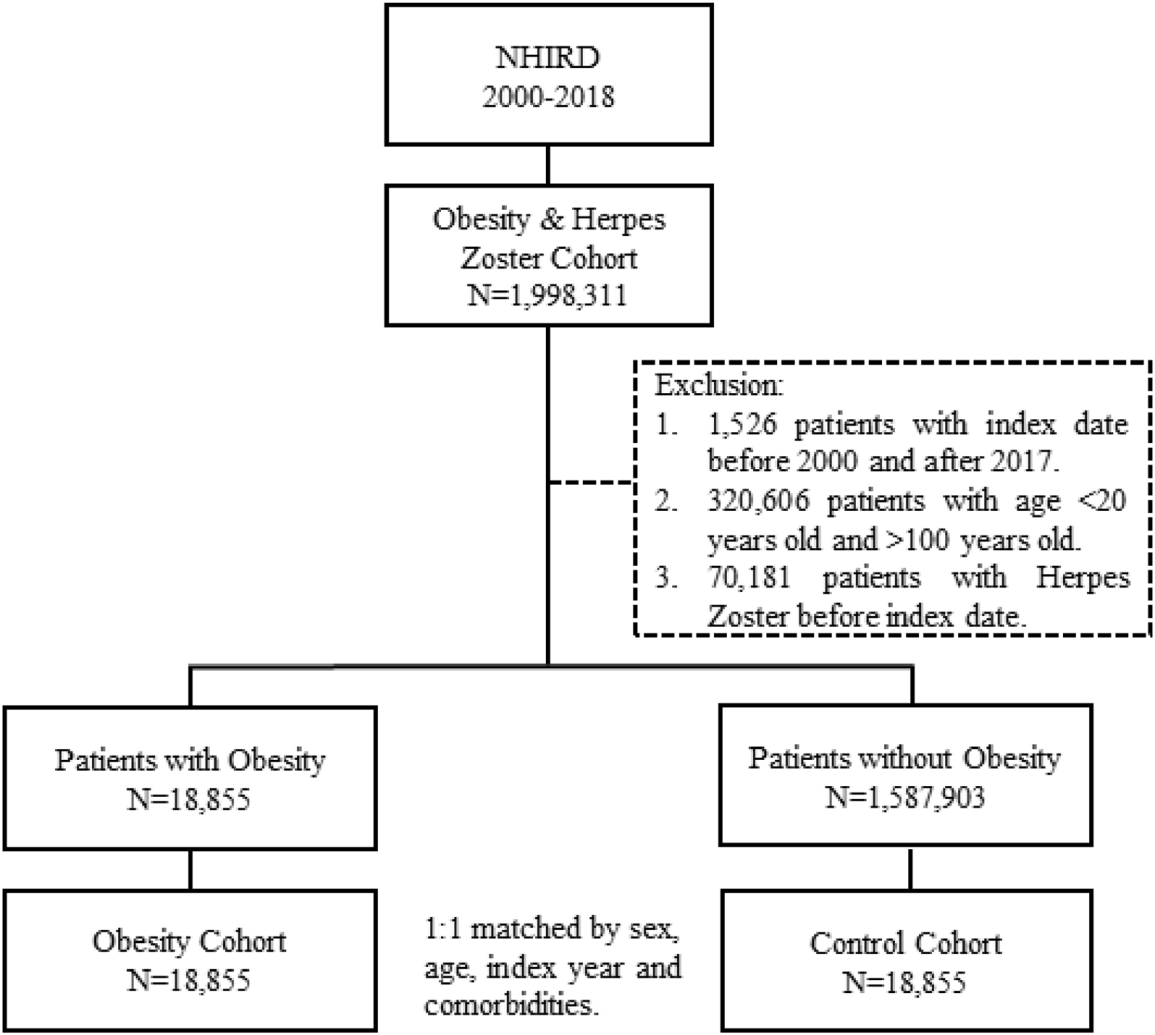
The flow chart of study sample selection from NHIRD Taiwan.

A flow chart of the patient’s selection from the NHIRD shown in Fig. 1. After propensity score matching, there were 18,855 patients in both the obesity and control cohorts. No significant differences in sex, age, and comorbidities were found between the two cohorts. Among the patients, the obesity and control cohorts had more than half women (58.35% and 58.37%, respectively). The mean age in the obesity and control cohorts were 43.31 (± 14.01) and 43.35 (± 14.06) years, respectively (Table 1). The median follow-up times in years for our cohorts were 7.47 for the control group and 7.76 for the obesity group. In the groups classified as non-morbid obesity and morbid obesity, the median follow-up times were 8.19 and 5.85 years, respectively.

**Table 1:** Baseline characteristics for individuals with and without obesity.

Variables	Obesity				*p*-value
	No (N = 18,855)		Yes (N = 18,855)		
	n	%	n	%	
Obesity type					
Non-morbid obesity	–	–	15,971	84.70	
Morbid obesity	–	–	2884	15.30	
Gender					
Female	11,005	58.37	11,002	58.35	0.97
Male	7850	41.63	7853	41.65	
Age, years					
20–29	3396	18.01	3403	18.05	1.00
30–39	4980	26.41	4981	26.42	
40–49	4361	23.13	4361	23.13	
≥ 50	6118	32.45	6110	32.41	
Mean ± SD^a^	43.35	14.06	43.31	14.01	0.75
Comorbidities					
DM	4617	24.49	4616	24.48	0.99
CKD	308	1.63	312	1.65	0.87
CAD	2832	15.02	2833	15.03	0.99
Cancer	412	2.19	415	2.20	0.92
Follow-up time, years					
Mean ± SD^a^	7.57	4.13	7.79	4.10	< 0.001
Median	7.47		7.76		

^a^t-test; *SD* standard deviation, *DM* diabetes mellitus, *CKD* chronic kidney disease, *CAD* coronary artery disease.

**Figure 2:**
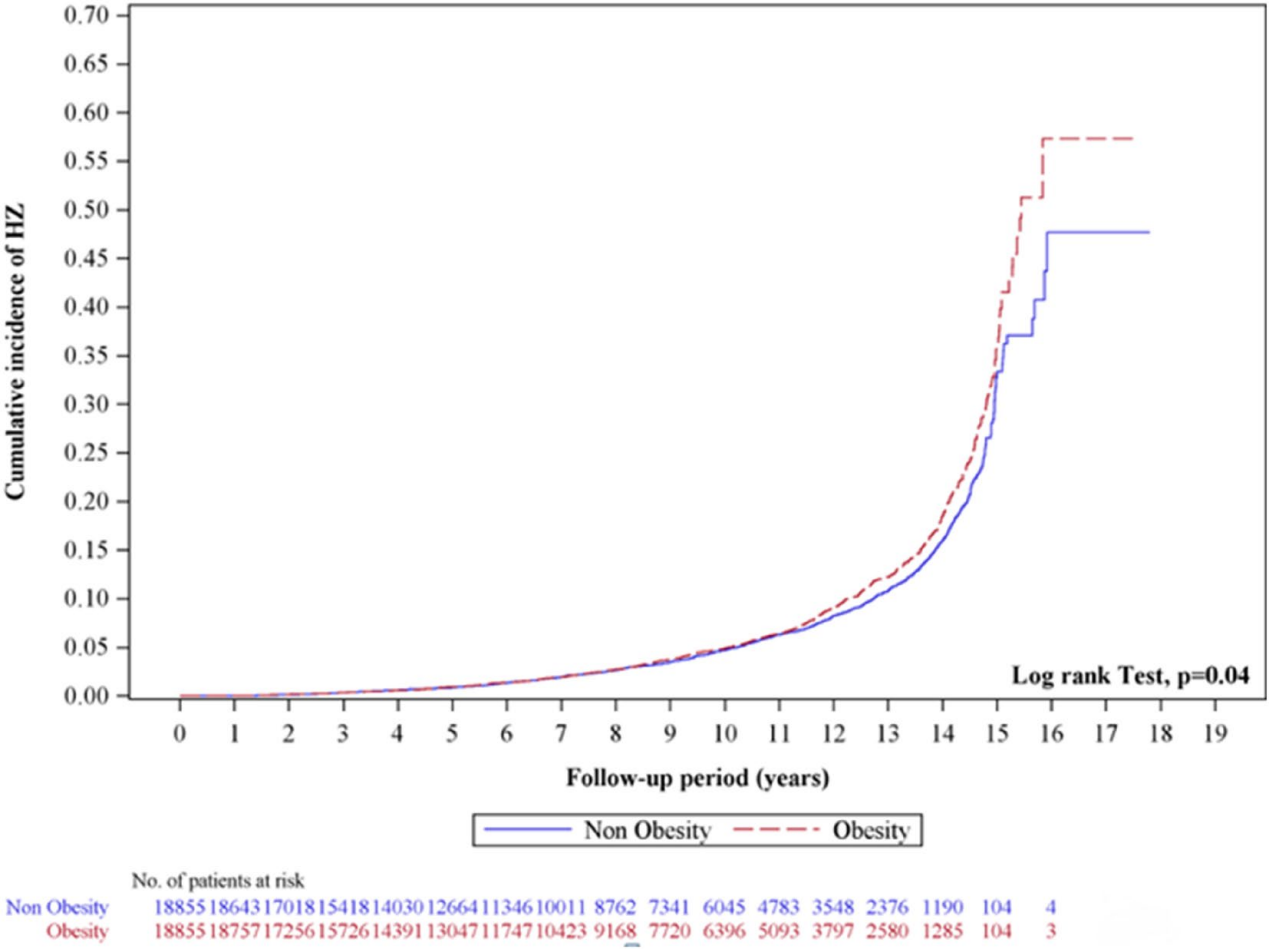
Kaplan–Meier curves of the cumulative incidence rate of herpes zoster during the follow-up period between obesity and control groups.

Table 2 presents events, person-year, IR, and HR for obesity, sex, age, and comorbidities in patients with HZ. The obesity cohort [adjusted HR (aHR) 1.09, 95% CI 1.00–1.19] had a significantly higher risk of HZ than the control cohort. Further analysis, the morbid obesity group (aHR 1.47, 95% CI 1.21–.77) had a significantly higher risk of HZ than the non-morbid obesity group. The age groups of 30–39 (aHR 1.35, 95% CI 1.12–1.62), 40–49 (aHR 1.87, 95% CI 1.57–2.23) and ≥ 50 (aHR 3.43, 95% CI 2.91–4.05) had a significantly higher risk of HZ than the age group of 20–29. The risk of HZ in patients with comorbidities was significantly higher than in those without comorbidities. The comorbidities included DM (aHR 1.18; 95% CI 1.07–1.30), CAD (aHR 1.31; 95% CI 1.17–1.46) and cancer (aHR 1.62; 95% CI 1.26–2.08). Figure 2 presents the cumulative incidence of HZ, it shows that the cumulative incidence of HZ in the obesity cohort was higher than that in the control cohort (*p* = 0.04).

**Table 2:** Incidences and hazard ratios of herpes zoster for individuals with and without obesity by age, gender and comorbidities.

Variables	Herpes zoster			cHR	(95% CI)	*p*-value	aHR	(95% CI)	*p*-value
	Event	PY	IR						
Obesity									
No	949	142,661	6.65	1.00	(reference)	–	1.00	(reference)	–
Yes	1115	146,810	7.59	1.13	(1.04, 1.24)**	0.005	1.09	(1.00, 1.19)*	0.041
Non-morbid obesity	990	128,194	7.72	1.00	(reference)	–	1.00	(reference)	–
Morbid obesity	125	18,616	6.71	1.17	(0.97, 1.41)	0.100	1.47	(1.21, 1.77)***	< 0.001
Gender									
Female	1333	175,904	7.58	1.00	(reference)	–	1.00	(reference)	–
Male	731	113,567	6.44	0.95	(0.87, 1.04)	0.251	1.01	(0.93, 1.11)	0.749
Age, year									
20–29	180	54,334	3.31	1.00	(reference)	–	1.00	(reference)	–
30–39	318	75,101	4.23	1.39	(1.15, 1.66)***	< 0.001	1.35	(1.12, 1.62)**	0.001
40–49	453	69,197	6.55	2.01	(1.69, 2.39)***	< 0.001	1.87	(1.57, 2.23)***	< 0.001
≥ 50	1113	90,839	12.25	4.03	(3.44, 4.72)***	< 0.001	3.43	(2.91, 4.05)***	< 0.001
Comorbidities									
DM									
No	1377	221,241	6.22	1.00	(reference)	–	1.00	(reference)	–
Yes	687	68,231	10.07	1.66	(1.51, 1.82)***	< 0.001	1.18	(1.07, 1.30)***	< 0.001
CKD									
No	2027	285,999	7.09	1.00	(reference)	–	1.00	(reference)	–
Yes	37	3473	10.65	1.96	(1.42, 2.71)***	< 0.001	1.26	(0.91, 1.75)	0.166
CAD									
No	1558	247,256	6.30	1.00	(reference)	–	1.00	(reference)	–
Yes	506	42,215	11.99	2.06	(1.87, 2.28)***	< 0.001	1.31	(1.17, 1.46)***	< 0.001
Cancer									
No	2000	284,399	7.03	1.00	(reference)	–	1.00	(reference)	–
Yes	64	5072	12.62	2.19	(1.71, 2.81)***	< 0.001	1.62	(1.26, 2.08)***	< 0.001

*DM* diabetes mellitus, *CKD* chronic kidney disease, *CAD* coronary artery disease, *PY* person-year, *IR* incidence rate, per 1000 person-years, *cHR* crude hazard ratio, *aHR* adjusted hazard ratio, adjusted for age, sex, index year and comorbidities, *CI* confidence interval; *p < 0.05, **p < 0.01, ***p < 0.001.

**Figure 3:**
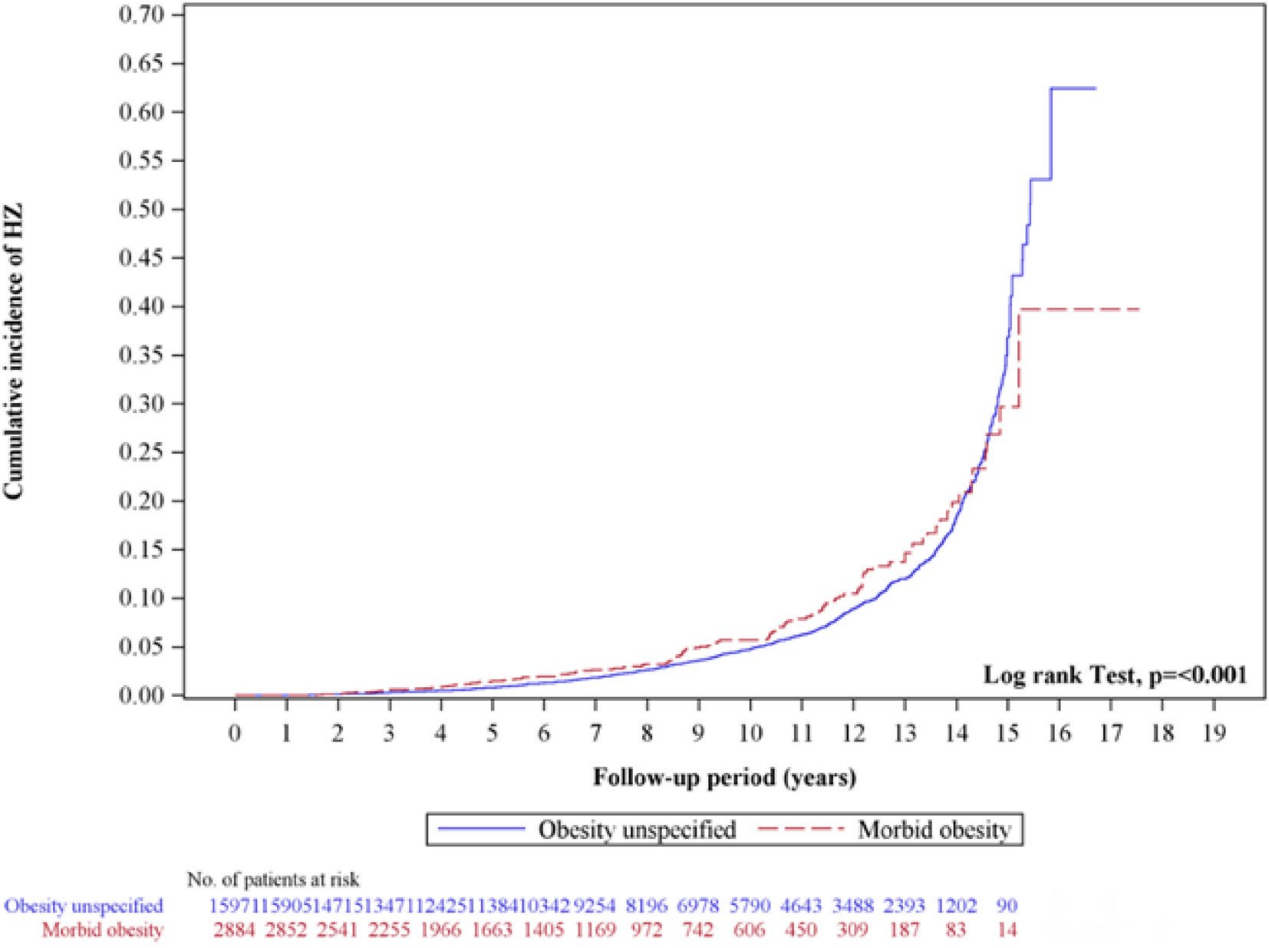
Kaplan–Meier curves of the cumulative incidence rate of herpes zoster during the follow-up period between non-morbid obesity and morbid obesity groups.

Table 3 shows the results of further stratification of obesity. In the obesity cohort, the risk of HZ in women (IR 8.10 vs. 7.04; aHR 1.12; 95% CI 1.00–1.24), those aged 30–39 years (IR 4.81 vs. 3.66; aHR 1.33; 95% CI 1.06–1.66), and without comorbidities (IR 6.09 vs. 5.13; aHR 1.18; 95% CI 1.05–1.33) was significantly higher than that of the control cohort. Table 4 presents the results from a more detailed stratification between non-morbid and morbid obesity cohorts. Within the morbid obesity group, the risk of HZ across different genders was significantly higher than in the control group. For females, the IR was 8.30 compared to 6.63 in the control group, with an aHR of 1.36 (95% CI 1.06–1.74). For males, the IR were 6.82 for the control group and 6.83 for the morbid obesity group, with an aHR of 1.66 (95% CI 1.24–2.23). Among the age groups 40–49 and ≥ 50, the risk of HZ in the morbid obesity cohort was also significantly higher compared to the non-morbid obesity cohort, with aHR of 1.59 (95% CI 1.10–2.30) and 1.58 (95% CI 1.18–2.12) respectively. Additionally, the analysis of comorbidities revealed that the morbid obesity cohort consistently displayed a higher risk of developing HZ compared to the non-morbid obesity cohort, irrespective of the presence of comorbid conditions. In Fig. 3, the Kaplan–Meier plots for non-morbid obesity and morbid obesity showed overlapping curves, suggesting a crossing lines phenomenon. To further investigate this, we subdivided the study population based on follow-up duration: less than 14 years and 14 years or more. This allowed us to separately evaluate the risk of developing HZ in the non-morbid and morbid obesity groups. We found a significant difference in the cumulative incidence of HZ between these groups during the shorter follow-up period (p < 0.001), with the morbid obesity group exhibiting a higher incidence compared to the non-morbid obesity group. However, no significant difference was observed in the groups with a follow-up of 14 years or more.

**Table 3:** Cox proportional hazards regression analysis for the risk of herpes zoster.

Variables	Obesity						cHR	(95% CI)	*p*-value	aHR	(95% CI)	*p*-value
	No			Yes								
	Event	PY	IR	Event	PY	IR						
Gender												
Female	613	87,015	7.04	720	88,889	8.10	1.15	(1.03, 1.28)*	0.01	1.12	(1.00, 1.24)*	0.04
Male	336	55,646	6.04	395	57,921	6.82	1.11	(0.96, 1.28)	0.16	1.06	(0.91, 1.22)	0.46
Age, year												
20–29	91	27,166	3.35	89	27,168	3.28	0.99	(0.74, 1.33)	0.95	0.98	(0.73, 1.31)	0.89
30–39	137	37,438	3.66	181	37,664	4.81	1.33	(1.06, 1.66)*	0.01	1.33	(1.06, 1.66)*	0.01
40–49	205	34,194	6.00	248	35,003	7.09	1.17	(0.97, 1.41)	0.09	1.18	(0.98, 1.42)	0.08
≥ 50	516	43,864	11.76	597	46,975	12.71	1.04	(0.93, 1.17)	0.48	1.03	(0.91, 1.16)	0.65
Comorbidities												
Without comorbidities	496	96,622	5.13	595	97,677	6.09	1.19	(1.06, 1.34)**	0.00	1.18	(1.05, 1.33)**	0.01
With comorbidities^a^	453	46,040	9.84	520	49,133	10.58	1.04	(0.92, 1.18)	0.54	1.01	(0.89, 1.14)	0.90
DM												
No	630	109,665	5.74	747	111,575	6.70	1.16	(1.05, 1.29)**	0.00	1.14	(1.02, 1.26)*	0.02
Yes	319	32,996	9.67	368	35,235	10.44	1.05	(0.90, 1.22)	0.56	1.01	(0.87, 1.17)	0.91
CKD												
No	937	141,121	6.64	1090	144,877	7.52	1.12	(1.03, 1.23)**	0.01	1.09	(1.00, 1.19)	0.05
Yes	12	1540	7.79	25	1932	12.94	1.51	(0.74, 3.07)	0.26	1.44	(0.7, 2.95)	0.32
CAD												
No	730	122,296	5.97	828	124,960	6.63	1.11	(1.00, 1.23)*	0.04	1.09	(0.98, 1.20)	0.11
Yes	219	20,366	10.75	287	21,850	13.14	1.16	(0.97, 1.38)	0.11	1.12	(0.94, 1.34)	0.19
Cancer												
No	914	140,426	6.51	1086	143,974	7.54	1.15	(1.05, 1.26)**	0.00	1.11	(1.02, 1.22)*	0.02
Yes	35	2236	15.65	29	2836	10.22	0.59	(0.36, 0.97)*	0.04	0.55	(0.33, 0.90)*	0.02

^a^Individuals with any comorbidity of diabetes mellitus (DM), chronic kidney disease (CKD), coronary artery disease (CAD), cancer were classified into the comorbidity group; *PY* person-year, *IR* incidence rate, per 1000 person-years, *cHR* crude hazard ratio, *aHR* adjusted hazard ratio, adjusted for age, sex, index year, diabetes, chronic kidney disease, coronary artery disease, and cancer, *CI* confidence interval; *p < 0.05, **p < 0.01, ***p < 0.001.

**Table 4:** Cox proportional hazards regression analysis for the risk of herpes zoster in different type of obesity.

Variables	Obesity						cHR	(95% CI)	*p*-value	aHR	(95% CI)	*p*-value
	Non-morbid obesity			Morbid obesity								
	Event	PY	IR	Event	PY	IR						
Gender												
Female	649	78,179	8.30	71	10,710	6.63	1.06	(0.83, 1.35)	0.66	1.36	(1.06, 1.74)*	0.02
Male	341	50,015	6.82	54	7906	6.83	1.38	(1.04, 1.84)*	0.03	1.66	(1.24, 2.23)***	< 0.001
Age, year												
20–29	72	21,997	3.27	17	5171	3.29	1.29	(0.76, 2.19)	0.35	1.30	(0.76, 2.20)	0.34
30–39	155	31,845	4.87	26	5819	4.47	1.27	(0.83, 1.93)	0.27	1.28	(0.84, 1.95)	0.25
40–49	215	31,105	6.91	33	3898	8.47	1.55	(1.07, 2.24)*	0.02	1.59	(1.10, 2.30)*	0.01
≥ 50	548	43,246	12.67	49	3729	13.14	1.62	(1.21, 2.18)**	0.00	1.58	(1.18, 2.12)**	0.00
Comorbidities												
Without comorbidities	521	84,674	6.15	74	13,002	5.69	1.16	(0.91, 1.48)	0.23	1.40	(1.10, 1.80)**	0.01
With comorbidities^a^	469	43,519	10.78	51	5614	9.08	1.39	(1.04, 1.87)*	0.03	1.57	(1.17, 2.11)**	0.00
DM												
No	655	97,152	6.74	92	14,423	6.38	1.20	(0.97, 1.50)	0.10	1.48	(1.18, 1.84)***	< 0.001
Yes	335	31,042	10.79	33	4193	7.87	1.24	(0.87, 1.78)	0.24	1.47	(1.02, 2.12)*	0.04
CKD												
No	968	126,485	7.65	122	18,392	6.63	1.16	(0.96, 1.41)	0.11	1.45	(1.20, 1.75)***	< 0.001
Yes	22	1708	12.88	3	224	13.39	2.23	(0.63, 7.86)	0.21	2.61	(0.69, 9.83)	0.16
CAD												
No	735	108,854	6.75	93	16,107	5.77	1.13	(0.91, 1.40)	0.28	1.42	(1.14, 1.76)**	0.00
Yes	255	19,340	13.19	32	2510	12.75	1.55	(1.07, 2.25)*	0.02	1.62	(1.11, 2.35)*	0.01
Cancer												
No	965	125,649	7.68	121	18,324	6.60	1.16	(0.96, 1.40)	0.13	1.45	(1.20, 1.76)***	< 0.001
Yes	25	2544	9.83	4	292	13.70	2.83	(0.93, 8.58)	0.07	2.36	(0.74, 7.52)	0.14

^a^Individuals with any comorbidity of diabetes mellitus (DM), chronic kidney disease (CKD), coronary artery disease (CAD), cancer were classified into the comorbidity group; *PY* person-year, *IR* incidence rate, per 1000 person-years, *cHR* crude hazard ratio, *aHR* adjusted hazard ratio, adjusted for age, sex, index year, diabetes, chronic kidney disease, coronary artery disease, and cancer, *CI* confidence interval; *p < 0.05, **p < 0.01, ***p < 0.001.

## Discussion

Extensive research has revealed a strong correlation between obesity and various chronic diseases, such as DM and CKD. Additionally, obesity has been found to be associated with musculoskeletal conditions like sciatica and endocrine-related disorders such as PCOS. It is noteworthy that these diseases have also been linked to the reactivation of HZ. This connection underscores the intricate interplay between obesity, its related health conditions, and their potential impact on the reactivation of HZ.

In a previous systematic review, Freemantle et al. reported a strong association between abdominal obesity and DM^16^. In addition, Lu et al. found that compared with the normal weight population, the odds ratios (OR) for DM were 1.55 and 1.85 for individuals with abdominal and compound obesity, respectively^17^. The authors suggested weight control to decrease the risk of DM^17^, particularly reducing waist circumference^16^. A population-based study, which used data from Taiwan’s NHI program, found that the incidence of HZ in patients with and without DM was 7.85 and 6.75 per 1000 person-years, respectively^18^. A recent meta-analysis found a similar HZ incidence among patients with DM, with an incidence of HZ of 7.22 and 4.12 per 1000 person-years in patients with and without DM, respectively^19^. Patients with DM had a higher risk of HZ than those without DM, and the relative risk (RR) was 1.38. Huang et al. suggested that HZ vaccination is necessary for patients with DM^20^.

Obesity increases the risk of CKD development. Chang et al.^21^ reported that compared with the normal weight population, the 5-year cumulative incidence of CKD in patients with overweight and obesity was 3.5 and 6.7 per 1000 person-years. A meta-analysis of 21 studies with an average follow-up period of 9.86 years revealed that patients with obesity had a higher risk of CKD than patients without obesity, and the RR was 1.81. The authors suggested that obesity can be used as a predictive factor for CKD development^22^. In patients with obesity, the mechanism of renal function impairment may be due to lipotoxicity and changes in adipose tissue secretions, resulting in renal inflammation, oxidative stress, and fibrosis^23^. A nationwide study found that the incidence of HZ in patients with predialysis CKD and patients without CKD was 8.76 and 6.27 per 1000 person-years, respectively. The authors reported that patients with predialysis CKD had a 1.4-fold increased risk of developing HZ compared with patients without CKD^24^. Dialysis was associated with a higher risk of HZ among patients with CKD^25^. Lin et al.^26^ presented the risk of HZ in patients with CKD treated with various therapies. They found that compared with the control group, the HR of HZ risk in patients with hemodialysis, peritoneal dialysis, and renal transplantation were 1.35, 3.61, and 8.46, respectively. Therefore, HZ vaccination is recommended for patients with CKD^25^.

Zhou et al.^27^ reported that BMI was significantly associated with an increased risk of sciatica, and the OR was 1.33. The authors also found that waist and hip circumference, total body fat mass, and fat percentage were associated with an increased risk of sciatica. A meta-analysis of 26 studies found that both overweight and obesity increased the risk of sciatica, and the OR were 1.12 and 1.31. In addition, the two factors also increased the risk of sciatica-related hospitalization, and the OR was 1.16 for overweight and 1.38 for obesity^28^. To prevent sciatica, weight control is recommended for patients with obesity^27,28^. In our previous study, patients with sciatica were 1.19-fold more likely to develop HZ compared with those without sciatica^14^. This may be because of immune system dysfunction in patients with chronic pain^29^.

Another meta-analysis found that women with PCOS had a higher rate of overweight and obesity. Compared with women without PCOS, women with PCOS had a RR of 1.95 for overweight, 2.27 for obesity, and 1.73 for central obesity^30^. PCOS typically manifests clinically after weight gain, and the main characteristics are hyperandrogenism, reproductive and metabolic dysfunction^31^. This is because the obese women with PCOS have a lower level of sex hormone-binding globulin and higher levels of total testosterone and fasting insulin^32^. Glueck et al.^33^ found that the symptoms of PCOS can be improved after 5–10% weight loss. Thus, weight control is critical for patients with PCOS^33^. In our previous study, we found that patients with PCOS had a higher risk of HZ development. The incidence of HZ in patients with and without PCOS was 3.92 and 3.17 per 1000 person-years, respectively. Patients with PCOS had a 1.26-fold higher risk of developing HZ than patients without PCOS^15^.

Previous studies have established a connection between DM, CKD, sciatica, PCOS, and the occurrence of HZ. However, our present study yielded distinct findings. We observed that among patients without any comorbidity, those with obesity alone exhibited a significantly higher risk of HZ compared to non-obese patients. This suggests that obesity could potentially act as a stressor that triggers the reactivation of HZ. These novel results highlight the role of obesity as a potential contributing factor in HZ reactivation, even in the absence of other comorbidities.

As this is retrospective study in nature, it is important to acknowledge its limitations. Firstly, the influence of weight loss medication on the study results cannot be fully accounted for. Since weight loss pills are not covered by the NHI in Taiwan, information regarding medication for weight loss is not available in the NHIRD. However, we believe that the impact of weight loss pills on the findings is minimal, considering the limited number of individuals who self-pay for such medication, thus allowing us to reasonably ignore any potential bias arising from weight loss pills. Second, previous studies have indicated that exercise can have a positive impact on the immune function of the human body^34^ and it may potentially influence the HZ occurrence. However, lifestyle factors, including exercise, are not available in the NHIRD. Therefore, the study is unable to account for the potential impact of exercise on the results. Thirdly, the NHIRD does not provide access to laboratory data such as glucose or creatinine levels. This absence of laboratory data limits the assessment of disease severity, which may potentially influence the study outcomes. Fourthly, since the study utilizes data from the NHIRD, the study population is identified using ICD diagnostic codes. The database does not explicitly record height and weight, making it impossible to calculate the body mass index. Although diagnoses may be made by physicians from various specialties, Taiwan's National Health Insurance Administration enforces a stringent audit system that penalizes inappropriate diagnoses and treatments. Therefore, the diagnoses in the study are considered reliable. Despite these limitations, it is worth noting that this study is based on nationwide data with a large sample size. Thus, it can still provide substantial evidence and serve as a valuable reference for clinicians and researchers in the field.

## Conclusion

Obese patients are at a higher risk of HZ development, especially in the patients with morbid obesity. Weight reduction is critical for preventing the onset of chronic diseases and decreasing the risk of HZ in patients with obesity.

## Data Availability

The data for this study were obtained from the National Health Insurance Research Database, which was provided by the Taiwan National Health Insurance Administration. However, due to the Personal Data Protection Act, the data cannot be publicly disclosed. Data for research purposes can be requested from the Taiwan National Health Insurance Administration (http://nhird.nhri.org.tw).
